# Fast entrainment of human electroencephalogram to a theta-band photic flicker during successful memory encoding

**DOI:** 10.3389/fnhum.2013.00208

**Published:** 2013-05-17

**Authors:** Naoyuki Sato

**Affiliations:** Department of Complex Systems, Future University HakodateHakodate-shi, Hokkaido, Japan

**Keywords:** subsequent memory paradigm, SSVEP, EEG oscillations, object-place binding, transient dynamics, entrainment of rhythms, computer simulations

## Abstract

Theta band power (4–8 Hz) in the scalp electroencephalogram (EEG) is thought to be stronger during memory encoding for subsequently remembered items than for forgotten items. According to simultaneous EEG-functional magnetic resonance imaging (fMRI) measurements, the memory-dependent EEG theta is associated with multiple regions of the brain. This suggests that the multiple regions cooperate with EEG theta synchronization during successful memory encoding. However, a question still remains: What kind of neural dynamic organizes such a memory-dependent global network? In this study, the modulation of the EEG theta entrainment property during successful encoding was hypothesized to lead to EEG theta synchronization among a distributed network. Then, a transient response of EEG theta to a theta-band photic flicker with a short duration was evaluated during memory encoding. In the results, flicker-induced EEG power increased and decreased with a time constant of several hundred milliseconds following the onset and the offset of the flicker, respectively. Importantly, the offset response of EEG power was found to be significantly decreased during successful encoding. Moreover, the offset response of the phase locking index was also found to associate with memory performance. According to computational simulations, the results are interpreted as a smaller time constant (i.e., faster response) of a driven harmonic oscillator rather than a change in the spontaneous oscillatory input. This suggests that the fast response of EEG theta forms a global EEG theta network among memory-related regions during successful encoding, and it contributes to a flexible formation of the network along the time course.

## Introduction

The medial temporal lobe has been thought to play an essential role in memory process of recent events (Scoville and Milner, 1957). More recently there is a consensus that the medial temporal lobe, especially the hippocampus, is thought to manage the episodic memory (O'Keefe and Nadel, 1978; Burgess et al., 2002; Morris et al., 2003; Eichenbaum and Lipton, 2008), where the episodic memory is declared by memory for personally experienced events set in a spatial-temporal context (Tulving, 1983). Neuroimaging studies have shown the association of the hippocampus with experimental models of the episodic memory such as object-location binding (Sommer et al., 2005), name-face association (Sperling et al., 2003), and word memory in a color-location context (Uncapher et al., 2006).

Theta band (4–8 Hz) power (Sederberg et al., 2003) and coherence (Fell et al., 2006) of intracranial electroencephalogram (EEG) in the medial temporal lobe are known to be stronger during successful memory encoding. Rutishauser et al. (2010) showed that phase locking of neuronal firing with local-field potential (LFP) theta is associated with subsequent memory performance. Scalp EEG theta is also found to be stronger during encoding for subsequently remembered items than for forgotten items (Klimesch et al., 1996; Weiss and Rappelsberger, 2000), and such subsequent memory effect was also found during performance of object-place associative memory (Summerfield and Mangels, 2005; Sato and Yamaguchi, 2007). This evidence suggests the existence of memory encoding processes associated with the dynamics of EEG theta.

By using EEG-functional magnetic resonance imaging (fMRI) simultaneous measurements, the authors showed that the memory-dependent scalp EEG theta power during encoding of the object-place associative memory correlated with blood-oxygen-level-dependent (BOLD) responses in multiple regions of the brain, including the anterior and posterior cingulate regions and the parahippocampal region (Sato et al., 2009) The task dependency of scalp EEG power-related regions is still under discussion; however, other EEG-fMRI studies have also shown that memory-dependent EEG theta associates with functional network consisting of multiple brain regions (Scheeringa et al., 2008; Michels et al., 2010; Hanslmayr et al., 2011). These evidences suggest that the multiple regions cooperate with EEG theta synchronization during successful encoding. However, a question still remains: What kind of memory-dependent neural dynamic organizes such a global network?

Neural entrainment is thought to be a key dynamic for forming a synchronized network among multiple functional units. In this dynamic, a coupling of two units having different oscillatory frequency and phases can form a synchronized activity in both units during a few oscillation cycles (Hoppensteadt, 1986; Izhikevich, 2007). The neural entrainment is also thought to be functional. For example, orientation-selective neurons in the cat primary visual cortex were shown to fire synchronously while receiving a coherent visual stimulus and this synchronization is expected to represent perceptual grouping (Gray et al., 1989). Motoneurons in the lamprey spinal cord are also thought to produce a regular locomotion pattern by using entrainment dynamic (Grillner, 2003). EEG is considered to reflect neural mass activity, and its temporal evolution was modeled with neuronal oscillators where the interaction of multiple regions was shown to result in a phaselocked oscillation between them (Jansen and Rit, 1995; David and Friston, 2003; David et al., 2005). Thus, the dynamic of the neural entrainment is expected to from an EEG synchronized network among distributed regions.

Here that the modulation of the EEG theta entrainment property during successful encoding was hypothesized to lead to a formation of an EEG theta synchronized network. To evaluate this hypothesis, a repetitive photic flicker in the theta band was used. The flicker is known to induce EEG predominantly in the occipital region at a specific frequency band given by the flicker (Herrmann, 2001), and the theta-band flicker induces EEG theta that can probe an EEG theta network indexed by temporal evolution of EEG against the flicker. Steady-state of visually evoked potentials (SSVEPs) elicited by the flicker was shown to correlate with visual perception (Srinivasan et al., 1999; Müller et al., 2003; Ellis et al., 2006; Kim et al., 2007; Parkkonen et al., 2008). On the other hand, for the current purpose, a transient response of EEG to the flicker is thought to be more important, as in this analogy of a driven harmonic oscillator; the time constant of the transient response is a function of the oscillator's intrinsic parameter, but its amplitude can be a function of the intensity of the external stimuli (See also section Theoretical Interpretation in Discussion). In experiments, the transient response of EEG to the flicker is known to reach a plateau in several oscillation cycles after the onset and the offset of the flicker (Harada et al., 1991; Müller et al., 1998). Müller et al. (1998) reported that a delay of the induced EEG power correlates with the attentional state of the subjects, but there is no report of a memory-dependent modulation of the transient response in the induced EEG.

The memory-dependent EEG is expected to be modulated by photic flicker with the following considerations. First, the flicker-induced EEG was found in the fronto-central region in addition to the occipital region (Harada et al., 1991; Silberstein et al., 2001; Ellis et al., 2006) in which region the subsequent memory effect was also observed (Klimesch et al., 1996; Weiss and Rappelsberger, 2000; Summerfield and Mangels, 2005; Sato and Yamaguchi, 2007). Second, a computer simulation of cortical network by the author predicted a possible interaction between flicker-induced EEG in the occipital region and pacemaker-driven EEG in the medial temporal lobe and that can be detected by scalp EEG in the frontal region (Sato, 2013). Third, the application of alpha-band flicker during memory encoding was shown to enhance subsequent memory recall (Williams, 2001). The flicker's frequency enhancing memory recall appeared specific within the alpha-band, thus the induced EEG itself was thought to influence the memory process, rather than that the flicker enhanced alert to visual stimuli.

In this study, a transient response of EEG to a short-duration photic flicker in the theta band was investigated during encoding, in relationship to subsequent recall. An object-place memory task in a virtual environment proposed by King et al. (2002) was used with a small modification where a subject with the damage in the hippocampus was shown to have difficulty performing the task. By using the object-place associative memory task, multiple regions including the hippocampus are expected to be activated during the encoding, and the theta-band flicker is expected to probe EEG theta dynamic in the functional network. When the EEG theta network is formed through neural entrainment among these regions, the transient response of EEG to the theta-band flicker should be a function of subsequent recall.

## Materials and methods

### Subjects

Sixteen participants with a mean age of 21.2 years (ranging from 20 to 23 years; 9 males) took part in the experiment. They showed no signs of neurological or psychiatric disorders and gave informed consent. All subjects were explicitly informed that flicker stimulation might lead to seizures in epileptics and reported that they had ever suffered from epilepsy. The protocol was approved by the Ethics Committee in Future University Hakodate.

### Stimuli

Snapshots of a virtual reality environment were presented on a 21-inch CRT monitor (Sony, CPD-G520) set to a 85-Hz refresh rate. Each snapshot was presented as 576 × 380 pixels (subtended 15.2 × 10°) against a gray background. A flicker was produced by five brief blinks of the snapshot at 7.08 Hz (= 1/12 frames^−1^), where each blink consisted of the entire snapshot becoming the background color with a duration of 47.1 ms (= 4 frames). During encoding, the five brief blinks were given twice, at 0.28 and 1.70 s after the stimulus onset.

The virtual environment was depicted as an 8 × 6 m square room implemented by using Simlink 3D Animation (MathWorks, USA). All sides of the room were surrounded by walls with directional cues consisting of a white-board, two doors and three windows. At the center of the room, a 2 × 2 m square table of a 0.6 m height was placed, on which an 5 × 5 array of filled-gray circular placeholders were presented. Objects that were put on placeholders were determined by natural packages such as bottles and containers (Dosch 3D, Dosch Design, Germany). By changing colors of the objects, each unique object appeared only once throughout the experiment. A viewer of height 1.5 m was displaced 2.5 m from the table, and its angular position was given by one of nine positions against a direction of the white board (0, 22.5, 67.5, 112.5, 157.5, 202.5, 247.5, 292.5, and 337.5°). Viewing direction of the viewer was to the head of the table. Before the measurement, all participants were familiarized with the environment by performing a voluntary-viewing task where the participants could continuously move the viewer in the environment around the table and change its viewing direction by pressing buttons on a keyboard.

### Procedure

**Figure 1:**
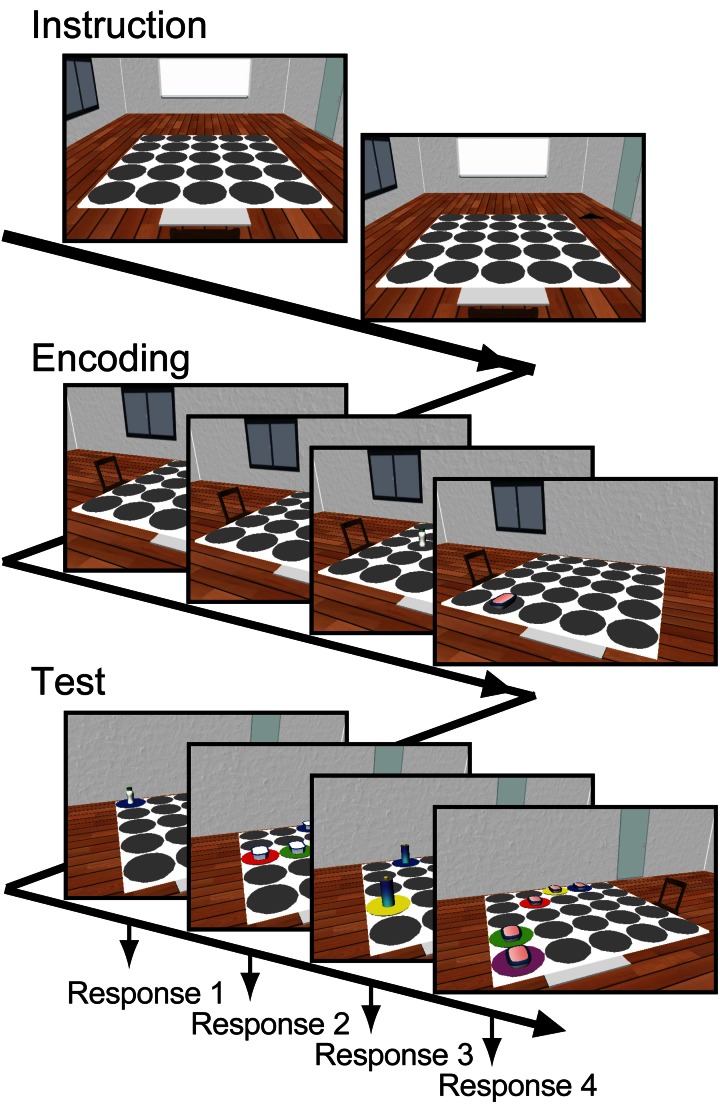
**Working memory task.** Each trial consists of instruction, encoding, and test phases. At the beginning of the trial, a starting viewing position in the environment was stated to the subject. Then, four snapshots viewed from the instructed position, including different objects located at different placeholders on the table, were presented for 3.1 s each. Two of the four snapshots were presented with flicker. The participants were asked to remember each object's location. During the test phase, the memory of each object's location was tested from a different viewing position by using a snapshot including the original object at the original location and four foils of identical objects at different locations. The participants were asked to report the correct object's location by using a keyboard.

Each participant performed 30 task-trials consisting of instruction, encoding, and test phases (Figure 1). At the beginning of the instruction, the viewer's location in the environment was always given by an angular position of 0°. One of the remaining eight angular positions was marked and then the viewer immediately moved to the marker position. This instruction allowed the participants to easily imagine where the viewer was located in the room. During encoding, four objects were placed on different randomly chosen placeholders. At the beginning of encoding, the participants were asked to focus on a fixation point at the center of the display. Then, each of the four objects was shown successively for 3.1 s each with an inter-stimulus interval of 1.5 s. Among them, a randomly selected two out of four were presented with the flicker procedure (“Flicker condition”) and the others remained without the flicker (“Constant condition”). The participants were asked to remember locations of each object. During the test, the memories for the locations of the objects were sequentially tested from a different viewpoint. At the beginning of the test, angular position of the viewer was immediately shifted 120 or 240° from the position during encoding. After a 5-s pause, the object at the original location and four identical objects at foil locations were presented. The placeholders under the five objects were differently colored. The participants were asked to press a colored key on the keyboard corresponding to the color of the correct placeholder of the original object within 7 s.

### EEG recording and analysis

EEG signals were acquired using a BrainVision amplifier (BrainProducts, Germany). The Ag/AgCl electrodes provided 26 EEG channels (a 10% standard system without Fp1 and Fp2) and four electro-oculography (EOG) channels. EEG data (0.01–100 Hz bandpass, 500 Hz sampling rate) were referenced to an FCz electrode during measurement and re-referenced to linked earlobes for analysis.

Ocular artifacts in EEG were corrected by using a regression subtraction method (Croft and Barry, 2000). Data segments of 500 ms during horizontal and vertical saccades were collected from the original data by eye inspection (300–650 segments), then regression coefficients between each EEG signal and horizontal and vertical EOGs were calculated by using the collected data. The topographic pattern of the coefficients at each electrode was checked by eye to be smoothly distributed over the scalp, in the range of 0–0.3 for vertical EOG and −0.3 to 0.3 for horizontal EOG. Then, the fixed regression coefficients were applied to a linear subtraction of the EOG data from the EEG data for all time-points.

Instantaneous frequency-energy characteristics at the flicker's frequency (7.08 Hz) for the corrected EEG data were analyzed with the Morlet wavelet transformation (width = 5; Tallon-Baudry et al., 1997). The wavelet log power during encoding in each trial that were subsequently recalled (“Hit”) or those that were not recalled (“Miss”) were compared by using a two-sample *t*-test. These comparisons were made separately for each electrode, each time point, and then averaged across all participants by using the inverse normal method (Lazar et al., 2002). The statistical level was corrected by using a surrogate procedure and false discovery rate (FDR) control. In the surrogate procedure, *t*-tests were performed to 1000 randomly shuffled data with trials for individual subjects and the statistical level was given by empirical distribution of 1000 averaged *t*-values across subjects. This procedure was used for the correction against the multicollinearly caused by the intercorrelation of the analytic window in the wavelet analysis. Multiple comparisons among the number of electrodes were corrected by using the FDR control (*q* = 0.05).

Phase locking of the wavelet phase during encoding was also evaluated by using a two-sample test for angular dispersion (Zar, 2009) compared between wavelet phases in the Hit trials and those in the Miss trials. In the calculation, wavelet phases of all *n* trials including both conditions at each electrode, *c*, and each time point, *t*, *a*^*c*^_*t*_ (*k*)(*k* = 1, 2, …, *n*), was transformed to angular distances from a mean angle ${\bar{a}}_{t}^{c}=\arg(\sum_{k=1}^{n}e^{ia_{t}^{c}(k)})$ as *d*^*c*^_*t*_(*k*) = |*a*^*c*^_*t*_(*k*) − *ā*^*c*^_*t*_| (ranged 0~ π), where the function arg(*z*) denotes the argument of a complex number *z*, and *i* denotes the imaginary unit. Then, angular distances in the Hit trials and those in the Miss trials were compared by using a two-tailed Mann-Whitney test. These comparisons were made separately for each electrode, each time point, and then averaged across all participants by using the inverse normal method. The statistical level was corrected by using a surrogate procedure and FDR control (*q* = 0.05).

Further, to evaluate possible influences of ocular artifact residuals, a clustering analysis was performed to show the interaction of temporal evolutions of wavelet phases at each electrode. First, the difference of mean wavelet phase in the Hit trials, *ā*^*c*^_*t*_, along with that in the Miss trials, ${\bar{b}}_{t}^{c}$, *p*^*c*^_*t*_ = *ā*^*c*^_*t*_ − ${\bar{b}}_{t}^{c}$, was calculated for each electrode, *c*, and each time point, *t*. The value was also used for display purposes. Then, the distance of temporal evolution of phase differences in two electrodes, (*p*^*c*_1_^_*t*_, *p*^*c*_2_^_*t*_) with time point, *t*, at each theta cycle (*t* = *kT, k* = 1, …, *M*), was given by:

$$d_{c_{1}c_{2}}=|1-r_{c_{1}c_{2}}|$$

where *t* denotes a theta cycle and *r*_*c*_1_*c*_2__ is an angular-angular correlation (Zar, 2009) given by,

$$r_{c_{1}c_{2}}=\frac{\sum_{k\;=\;1}^{M-1}\sum_{l\;=\;k\;+\;1}^{M}\sin\;(p_{kT}^{c_{1}}-p_{lT}^{c_{1}})\sin\;(p_{kT}^{c_{2}}-p_{lT}^{c_{2}})}{\begin{array}{l} \sqrt{\sum_{k\;=\;1}^{M\;-\;1}\sum_{l\;=\;k\;+\;1}^{M}\sin^{2}\left(p_{kT}^{c_{1}}-p_{lT}^{c_{1}}\right)}\\ \;\sum_{k\;=\;1}^{M\;-\;1}\sum_{l\;=\;k\;+\;1}^{M}\sin^{2}\left(p_{kT}^{c_{2}}-p_{lT}^{c_{2}}\right) \end{array}}.$$

Base on this distance metric, temporal evolution of phase differences in all electrodes was clustered by using a traditional Ward's method (Everitt et al., 2001).

## Results

### Task performance

All of the participants except for one showed a significant hit rate of recall for objects' locations [mean hit rate = 59.4% (chance level = 25%), *Z* = 41.8, *p* < 0.001]. All of the participants showed no significant difference between hit rate in the Flicker condition and that in the Constant condition (*Z* = 1.28, n.s.). This result is contrast with the evidence by Williams (2001) where the alpha-band flicker was shown to enhance subsequent memory recall. No participants reported any discomfort related to the flicker. The data of the one participant showing a hit rate below the chance level were excluded in the following analysis.

### EEG power during constant condition

**Figure 2:**
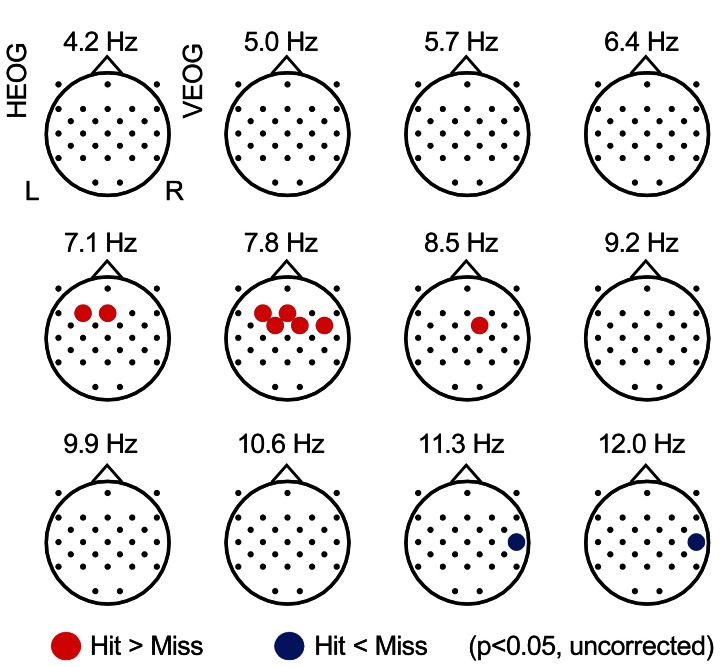
**Subsequently-recall related EEG power.** Topographical maps showing average *p*-values comparing EEG power during encoding of subsequently recalled items (“Hit” condition) with that of not recalled items (“Miss” condition). Each point represents the electrode locations, in which the color indicates the change: red represents an increase in EEG power (*p* < 0.05, uncorrected) and blue represents a decrease of EEG power (*p* < 0.05, uncorrected). HEOG, horizontal electro-oculography; L, left; R, right; VEOG, vertical electro-oculography.

Figure 2 displays the topographical pattern of the subsequent memory effect of each frequency power during the Constant condition, represented by *p*-value averaged across all participants (uncorrected for multiple comparison with electrodes). An increase of EEG power was found at theta range of 7.1–8.5 Hz, in the frontal region (with a maximum at the F3 electrode of 7.1 Hz, *p* = 0.029, uncorrected) and an EEG power decrease was also found in the alpha range of 11.3–14.9 Hz in the right temporal region (with a maximum at T8 of 12.0 Hz, *p* = 0.016, uncorrected). These effects did not appear significant with FDR control. However, these spectral patterns in relationship with subsequent recall agree with previous reports (Weiss and Rappelsberger, 2000; Sato and Yamaguchi, 2007; Sato et al., 2009; Hanslmayr et al., 2011).

### Transient response of flicker-induced EEG power

**Figure 3:**
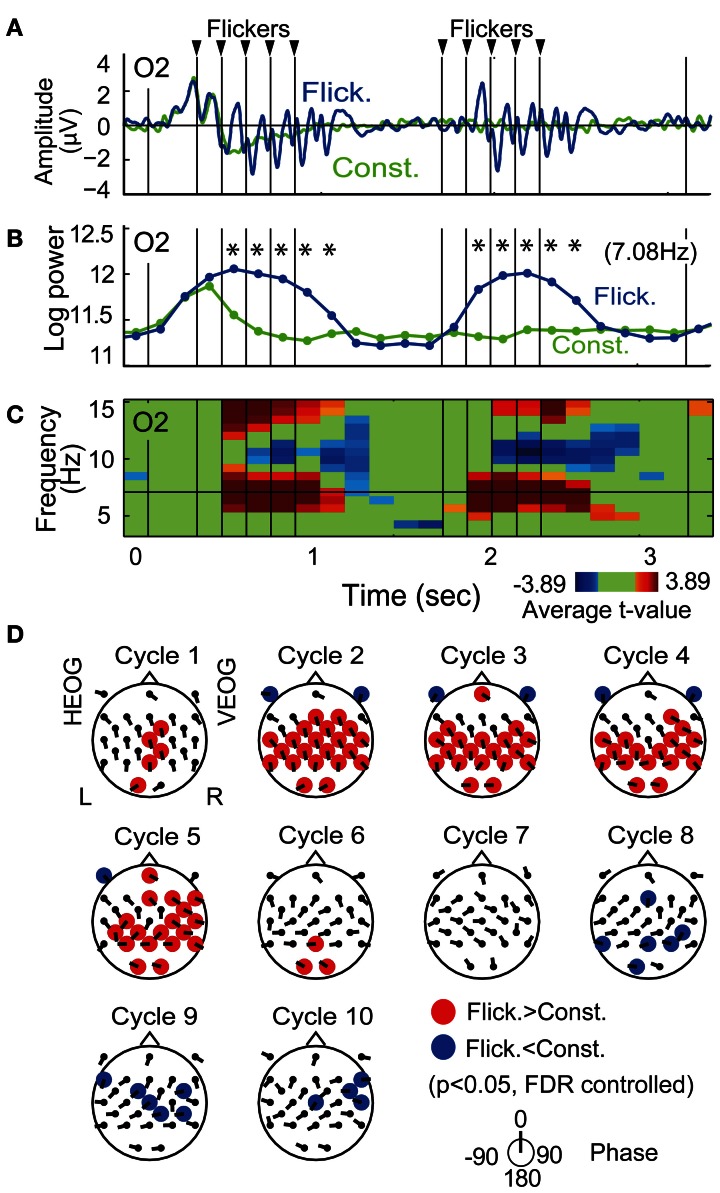
**Flicker-induced EEG. (A)** Temporal evolution of EEG signal during encoding at an O2 electrode averaged across all participants. Blue and green indicate EEG signals in the Flicker and Constant conditions, respectively. For display purposes, the EEG signals were band-passed between 3 and 20 Hz by using a Butterworth filter. Triangles at the upper part of the plot indicate timings of flicker. **(B)** Temporal evolution of EEG power at the flicker's frequency. Asterisks indicate significant difference of EEG power in Flicker condition with that in Constant condition (*p* < 0.05). **(C)** Time-frequency plot of average *t*-values comparing EEG power in Flicker condition with that in Constant condition. Red represents significant increase of EEG power and blue represents significant decrease of EEG power. Green represents no significant difference. **(D)** Topographical maps of average *p*-value at each cycle showing difference of EEG power in Flicker condition with that in Constant condition. Red represents significant increase of EEG power and blue denotes significant decrease of EEG power (*p* < 0.05, FDR controlled). Direction of arrow at each electrode location indicates a mean phase of EEG in Flicker condition against the flicker timings.

Figure 3A shows the temporal evolution of flicker-induced EEG at an O2 electrode. Oscillation of the potential appears in relationship with the onset and the offset of the flicker, with a delay of several hundred milliseconds. Temporal evolution of EEG wavelet power at the flicker's frequency (Figure 3B) shows a significant increase during a period from the second theta cycle after the onset of the flicker to the second theta cycle after the offset of the flicker (*p* < 0.05, FDR controlled). Figure 3C shows a time-frequency plot of average *t*-values comparing EEG power in the Flicker condition and that in the Constant condition at the O2 electrode. A significant increase of EEG power was found at the flicker's frequency and its second harmonic. A significant decrease of EEG power was also found at the alpha band. Importantly, no significant increase of EEG power was found in the lower frequency band and it suggests that residuals of ocular artifacts did not dominate EEG power in the Flicker condition. The statistical values in the first five and the latter five flickers appear similarly across the time period; therefore these were averaged in the following analysis. In individual analysis, data of two participants did not show any significant increase in the flicker-induced EEG power, hence these were excluded and data from the remaining thirteen participants were used in the following analysis.

Figure 3D displays the topographical pattern of the flicker-induced EEG power at each cycle represented by *p*-values averaged across all participants. A significant increase of EEG power was dominantly found in the occipital region and also found in a widely distributed in other regions (*p* < 0.05, FDR controlled). A significant decrease of EEG power was found in a widely distributed region after the flicker offset (*p* < 0.05, FDR controlled). This effect of power decrease after flicker offset was also reported with the use of a 10-Hz flicker (Harada et al., 1991).

On the other hand, mean EEG phases at each cycle and each electrode, which are shown by the directions of arrows at each electrode location in Figure 3D, did not appear uniformly over the scalp. This suggests that the EEG at each electrode were locked at different phases of the flicker. Neither volume conduction of the occipital EEG nor contamination of a flicker-related activity in the reference electrode are considered to be likely primary reasons for the induced-EEG power widely distributed over the scalp.

### Subsequent memory effect in the transient response of EEG power

**Figure 4:**
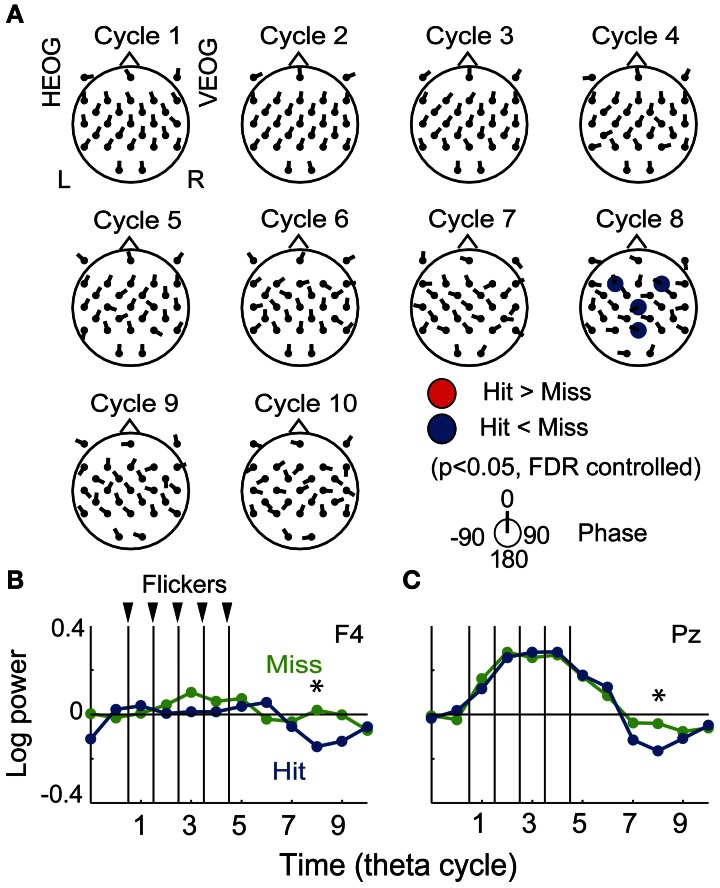
**Subsequent recall-related flicker-induced EEG power. (A)** Topographical maps of average *p*-value at each cycle showing difference between EEG power in the Hit condition and that in the Miss condition (*p* < 0.05, FDR controlled). Direction of arrow at each electrode location indicates a mean EEG phase in the Hit condition against that in the Miss condition. **(B,C)** Temporal evolution of EEG power against a mean EEG power in Constant condition averaged over all participants (at F4 and P4 electrodes). Blue and green represent EEG power in the Hit condition and that in the Miss condition, respectively. Asterisks indicate significant difference between EEG power in the Hit condition and that in the Miss condition.

The fronto-central region was found to show both the subsequently memory effect (Figure 3) and the flicker-induced EEG power increase (Figure 4), thus the region was expected to demonstrate a flicker-related subsequent memory effects. The resign of interest was set to seven electrodes in the frontal region (FC5, FC1, FC2, FC6, F3, Fz, and F4) and applied for the FDR control in the following analysis. Figure 4A displays the topographical pattern of average *p*-values comparing EEG power in the Hit condition to that in the Miss condition at each cycle. The *p*-values out of the fronto-central region were shown for visualization purpose by using the same significance level. No significant increase of EEG power was found, while a significant decrease of EEG power was found at the eighth cycle (the fourth cycle after the flicker offset) in the frontal region (at F3 and F4 electrodes, *p* = 0.05, FDR controlled), but no significant increase of EEG power was found. Decrease of EEG power in the central region (at a Cz electrode) and the parietal region (at a Pz electrode) were also shown in the figure. Figure 4B show temporal evolutions of EEG power in the frontal region (at a F4 electrode). Interestingly, EEG power in the Hit condition was found to quickly decrease below the baseline after the flicker offset, while that in the Miss condition tended to remain after the flicker offset. This property also appeared similarly in the parietal region (Figure 4C). On the other hand, it is difficult to find a clear relationship between the mean EEG phase and the subsequent memory effect in EEG power from the topographical pattern of mean EEG phase in the Hit condition against that in the Miss condition (Figure 4A).

### Subsequent memory effects in the transient response of EEG phase locking

**Figure 5:**
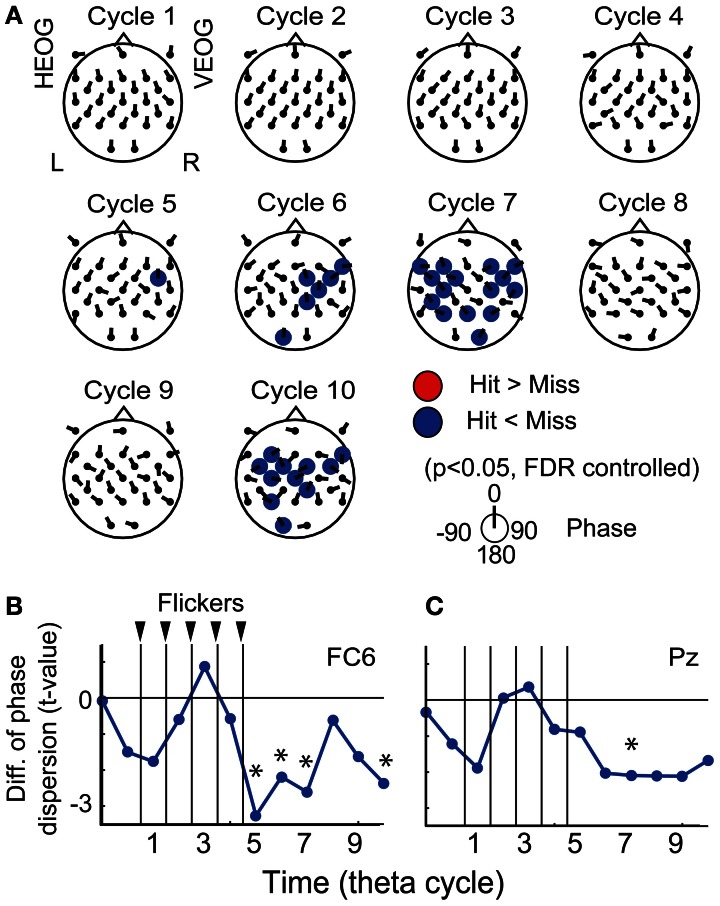
**Subsequent recall-related EEG phase locking. (A)** Topographical maps of average *p*-values at each cycle showing differences between EEG phase locking in the Hit condition and that in the Miss condition. Direction of arrow at each electrode location indicates a mean EEG phase in the Hit condition against that in the Miss condition. **(B,C)** Temporal evolution of average *t*-values comparing phase locking in the Hit condition with that in the Miss condition (at FC6 and Pz). Asterisks indicate significant difference between phase locking in the Hit condition and that in the Miss condition.

Figure 5A displays the topographical pattern of average *p*-values comparing EEG phase locking in the Hit condition to that the in the Miss condition at each cycle. No significant increase of phase locking was found, while a significant decrease of phase locking was found at the fifth-seventh and the tenth cycles after the flicker onset in the frontal region (*p* < 0.05, FDR controlled). This indicates that phase locking in the Hit condition after the flicker offset (the fifth-seventh and the tenth cycles) is weaker than that in the Miss condition. Temporal evolution of the average *t-value* in the fronto-central region (at a FC6 electrode; Figure 5B) clearly appears to decrease below the baseline after the flicker offset. These results clearly show that the phase locking to the flicker is quickly broken after the flicker offset in the Hit condition. Moreover, the decrease of phase locking was found in more widely distributed regions and the temporal evolution of the averaged *t-value* in the parietal region (at a Pz electrode) appeared similar to that in the fronto-central region.

### Clustering analysis for the memory-dependent temporal evolution of EEG phase

**Figure 6:**
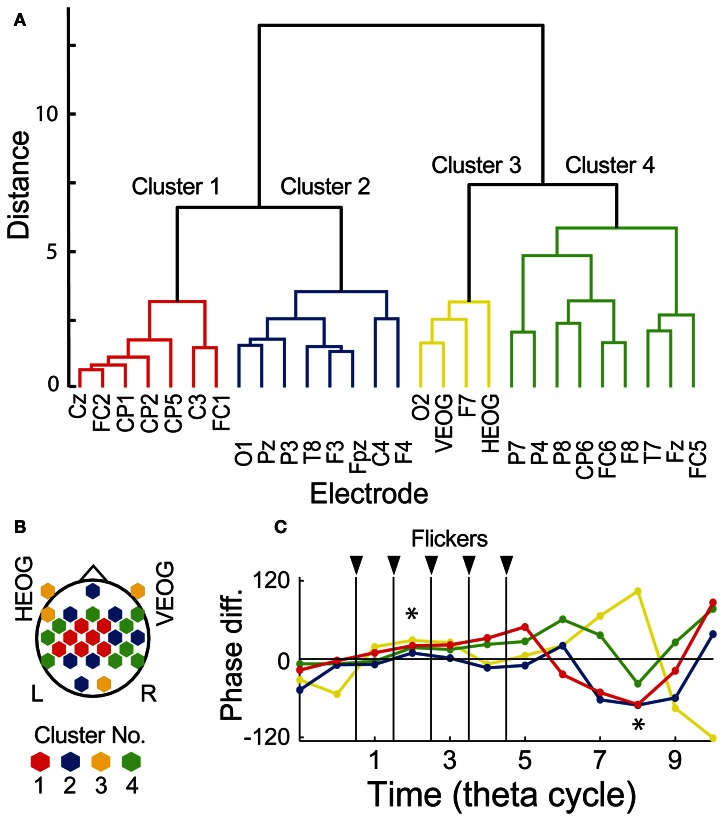
**Clustering analysis by using temporal evolution of EEG phase. (A)** Dendrogram showing resultant clusters of electrodes by using temporal evolution of their mean EEG phase in the Hit condition against that in the Miss condition. **(B)** Topographical map showing electrode locations in each cluster. **(C)** Temporal evolution of mean EEG phase in the Hit condition against that in the Miss condition in each cluster. Red, blue, yellow, green indicate cluster 1, 2, 3, and 4, respectively. Asterisks indicate time periods in which mean EEG phase in the Hit condition against that in the Miss condition of all electrode showed significant difference from 0° (*p* < 0.05, Bonferroni corrected).

To evaluate possible influences of ocular artifact residuals, interaction of EEG signals in relationship with subsequent recall was evaluated by using a hierarchical clustering analysis of temporal evolution of mean EEG phase in the Hit condition against that in the Miss conditions, already shown in Figures 4A, 5A as arrows at each electrode location. Figure 6A shows a result of the hierarchical clustering of electrodes where the temporal evolution of the phase is divided into four clusters. Figure 6B shows a topographical pattern of electrodes in each cluster. The electrodes in the first cluster (shown by red) were located at the central region and those in the second and the forth clustered (shown by blue and green, respectively) were located to surround the central region. The third cluster (shown by yellow) includes EOG electrodes. Figure 6C shows temporal evolutions of mean EEG phase in the Hit condition against that in the Miss condition in each cluster. When a set of mean EEG phase of all electrode was statistically analyzed at each cycle, the phase at the second and the eighth cycles were found to be significantly different from 0° (one-sample test for mean angle, *N* = 28, *p* < 0.05, Bonferroni corrected for the number of cycles). These results agree with the subsequently memory effect in EEG power and phase locking shown in Figures 4, 5. Among clusters, the first cluster appears to correspond with the results of EEG power and phase locking in terms of the topographical pattern and the temporal evolution of the EEG phase.

## Discussion

**Figure 7:**
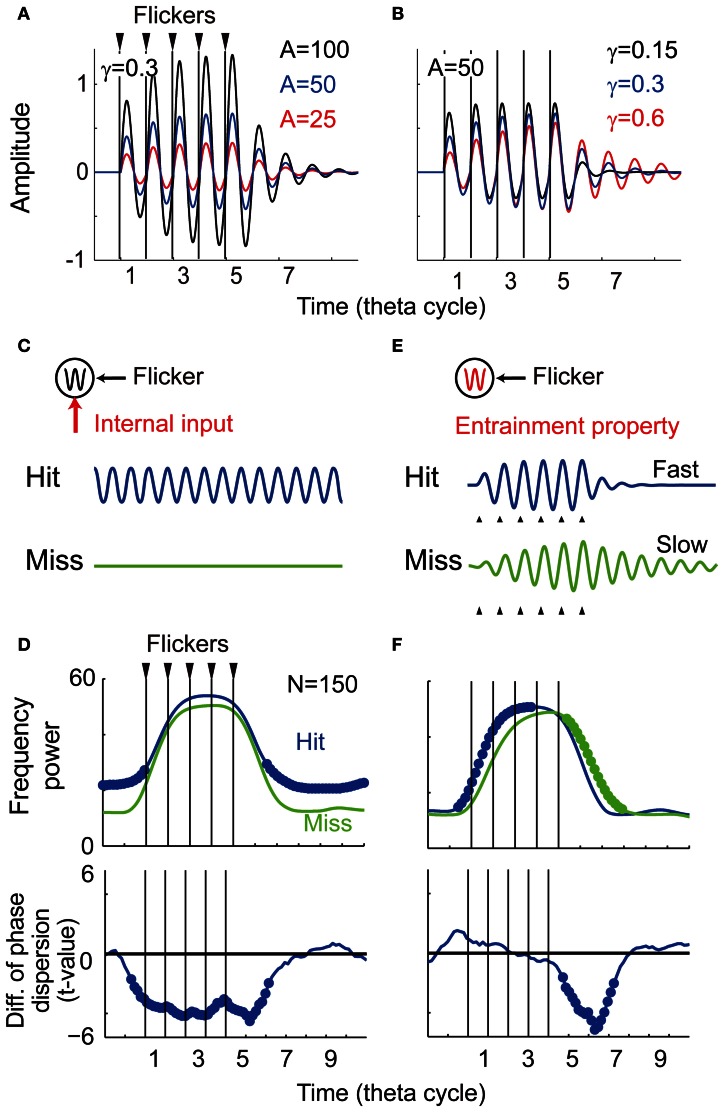
**Computational simulation. (A)** Temporal evolution of potentials with different intensity of the input (α = 25, 50, and 100). **(B)** Temporal evolution of potentials with different damping coefficient of oscillator (γ = 0.15, 0.3, and 0.6). **(C)** The first model assuming a memo-dependent oscillatory input in which phase is independent to the phase of the flicker. **(D)** Simulation results of the first model showing averaged spectral power and phase locking (*N* = 150, *p* < 0.05). Bold line indicates significant difference between the Hit condition and the Miss condition. **(E)** The second model assuming memory-dependent damping coefficient that is smaller during successful encoding (the Hit condition: γ = 0.3, the Miss condition: γ = 0.6). **(F)** Simulation results of the second model showing averaged spectral power and phase locking (*N* = 150, *p* < 0.05).

The present results showed a memory-related transient response of the EEG to a theta-band photic flicker. The flicker-induced EEG power at the fronto-central region was found to quickly decrease below the baseline after flicker offset during successful encoding (Figure 5). A similar effect was also found in the phase locking over the distributed region, which appeared to quickly decrease after the flicker offset during successful encoding (Figure 6). In addition, the clustering analysis of the temporal evolution of the memory-dependent EEG phase at each electrode showed a topographic pattern that was different from that of the EOG electrodes (Figure 7). These results showed the transient response of the EEG to the flicker changes during successful encoding. This modulation in the transient response is thought to be implemented by the change in the entrainment property of the EEG. The implication of these results will be discussed below.

### Memory-dependent transient response of EEG to the theta-band flicker

Temporal evolution of the flicker-induced EEG after the onset and the offset of the flicker are shown to appear in a duration of a several hundred milliseconds (Harada et al., 1991; Müller et al., 1998). Müller et al. (1998) reported that a delay in the induced EEG power to the flicker onset correlates with the attentional state of the participants, and this phenomenon was interpreted as a visual pathway facilitation. On the other hand, in the current results, the memory-dependent transient response of the flicker-induced EEG after the flicker onset was found, but the transient response after the flicker offset was more clearly observed. The visual pathway facilitation most likely explains the faster response after the flicker onset, but it does not directly associate with a faster response after the flicker offset. Another interpretation is necessary for the current results, and that will be discussed in the next section by using computer simulation.

SSVEP elicited by photic flicker during performance of the memory task has been shown to associate with memory performance (Silberstein et al., 2001; Ellis et al., 2006). However, in the current results, the flicker-induced EEG after a few oscillation cycles, which could include some components of SSVEP, was not found to be memory-dependent. This could be explained by a difference in data analysis. In the SSVEP analysis, more than several flicker-induced potentials are usually averaged and that produces a high signal-to-noise ratio (Regan, 1989). The short-duration flicker used in this study does not produce a period for stable flicker-induced oscillations, while it does have an advantage in reducing discomfort of the participants and, importantly, the ability to probe transient responses of the EEG, which would be different information from that in the SSVEP analysis.

Repetitive transcranial magnetic stimulation (rTMS) is also a good tool to probe the temporal property of EEG dynamic and further show the causality between brain waves and function (Thut and Miniussi, 2009). Johnson et al. (2010) compared influences of rTMS and the photic flicker, and reported that the influence of rTMS on an artifact-corrected EEG was subtle in comparison to that of the photic flicker. This does not reject a possible application of rTMS to probe neural entrainment in an EEG theta synchronized network; however, the photic flicker is thought to be a good tool for the current purpose.

### Theoretical interpretation

The current results suggested that intrinsic modulation of EEG theta dynamics associates with the successful memory encoding, rather than that the flicker-induced EEG theta directly contributes to the memory encoding. However, in the level of neuronal dynamics, some possibilities remain for interpreting the current results, i.e., enhancement of each theta-band phrasemaker in a region and theta-band EEG resonance in the network can be available for the interpretation. In this section, these possibilities will be discussed by using a computational simulation.

Here a computational model of EEG is used to investigate what change in model parameter can explain the current results. Many models have been shown the contribution of theta-band dynamics in synaptic plasticity (Jensen and Lisman, 1996; Hasselmo et al., 2002; Sato and Yamaguchi, 2003), while the current model is intended to phenomenologically evaluate temporal dynamics of EEG without synaptic plasticity. Spontaneous EEG and event-related potential has been modeled by neural mass oscillators (Jansen and Rit, 1995; David and Friston, 2003; David et al., 2005), while the temporal evolution of the flicker-induced EEG can be phenomenologically modeled by a driven harmonic oscillator, in which amplitude gradually follows to the onset and the offset of a periodic driving input. The driven harmonic oscillator is one of simplest models for describing oscillatory phenomena, and it is used here to describe the current observation. The oscillator is described by a second order differential equation given by,

$$\frac{d^{2}x}{dt^{2}}+2\gamma \frac{dx}{dt}+\omega^{2}x=\frac{I(t)+\varepsilon (t)}{\gamma }$$

with

$$I(t)=\{\begin{array}{cc} \alpha \delta (t^{\prime }) & \left(t^{\prime }=\frac{2\pi n}{\omega },n=1,\ldots ,5\right)\\ 0 & \left(\text{otherwise}\right) \end{array}$$

where *x* denotes the potential, γ denotes a damping coefficient, ω denotes a constant for angular frequency, *I*(*t*) is a flicker input with intensity of α, δ(*t*) is the delta function and ε(*t*) is a Gaussian perturbation with variance 1. In the absence of the input (*I*(*t*) = ε (*t*) = 0), the temporal evolution of the potential becomes a damping oscillation given by,

$$x(t)\sim X_{0}e^{-\gamma t}\cos\;\omega t$$

under a condition of *x*(0) = *X*_0_, $\frac{dx(0)}{dt}=0$, and γ << ω. Note that the time constant of the damping oscillation just depends on the damping coefficient and is independent to the other conditions.

To show the parameter dependency of the oscillator's behavior, here the temporal evolution of the potential was evaluated without perturbation (ε(*t*) = 0). Figure 7A shows three temporal evolutions of the potential for different intensities of the flicker (α = 25, 50, and 100). The amplitude of the oscillation increases for larger intensities of the flicker, while the time constant of the oscillation appears constant. Figure 7B shows three temporal evolutions of the potential for different damping coefficients (γ = 0.15, 0.3, and 0.6). In contrast with the above, the amplitude of the oscillation appears constant, but the time constant of the oscillation is faster for the smaller damping coefficient. These results of the simulations suggest that the time constant of the induced oscillation is a function of the damping coefficient, but not a function of the intensity of the flicker.

The following two probable mechanisms were evaluated to reproduce the current experimental observation. The first model assumes a memory-dependent oscillatory input, in which frequency is identical and phase is independent to the phase of the flicker. When the oscillatory input is assumed to be strong during successful encoding (Figure 7C), a re-entrainment of the potential to the oscillatory input is thought to result in a quick decrease of the induced oscillation after the flicker offset. Figure 7D shows averaged spectral power and phase locking of simulated data (*N* = 150). In contrast to the experimental observation, the averaged power appeared spontaneously large during the non-flicker period. The phase locking during the non-flicker period also appeared continuously weaker in addition to the weak phase locking just after the flicker offset. These results do not characterize the experimental findings and some other mechanism should be considered.

The second model assumes a smaller damping coefficient during successful encoding that creates a faster response of the induced EEG (Figure 7E). Figure 7F shows the result of averaged spectral power and phase locking of simulated data (*N* = 150). The significant negative difference in power and phase locking were obtained after the flicker offset, as in the current experimental result (see Figures 4, 5). In contrast to the experimental results, the averaged spectral power after the flicker onset was found to significantly increase. This asymmetric effect between the onset and offset of the flicker is a nature of the harmonic oscillator as demonstrated in Figure 7B. In the experimental results, some changes during the flicker onset, such as ocular artifacts, may be thought to disturb a statistical detection of that effect. On the other hand, no significant change in the phase locking after the flicker onset agrees with the experimental results. This is reasoned by a difficulty in statistical detection of that effect after the flicker onset where variance of EEG phase before the flicker onset is larger than that before the flicker offset. These results support the modulation of the entrainment property during encoding in relationship to subsequent recall. The neuronal mechanism of the change in the damping coefficient is still unclear, but it could associate with a strength of inhibition among a population activity of interacting spontaneous oscillators (Sato, 2013).

Here the transient response of EEG theta to a theta-band photic flicker during memory encoding was demonstrated to predict subsequently memory recall. i.e., EEG theta was found quickly desynchronized after the offset of the flicker during successful encoding. According to the computational simulations, this is interpreted as a smaller time constant (i.e., faster response) of a driven harmonic oscillator during successful encoding. It suggests that the fast response in EEG theta forms a global EEG theta network among memory-related regions during successful encoding.

### Conflict of interest statement

The author declares that the research was conducted in the absence of any commercial or financial relationships that could be construed as a potential conflict of interest.
